# Shared songs are of lower performance in the dark-eyed junco

**DOI:** 10.1098/rsos.160341

**Published:** 2016-07-27

**Authors:** Gonçalo C. Cardoso, Jonathan W. Atwell

**Affiliations:** 1 CIBIO—Centro de Investigação em Biodiversidade e Recursos Genéticos, Universidade do Porto, Campus Agrário de Vairão, 4485-661 Vairão, Portugal; 2 Department of Biology, Indiana University, 1001 E. 3rd Street, Bloomington, IN 47405, USA

**Keywords:** animal communication, cultural evolution, improvisation, invention, social learning, song performance

## Abstract

Social learning enables the adjustment of behaviour to complex social and ecological tasks, and underlies cultural traditions. Understanding when animals use social learning versus other forms of behavioural development can help explain the dynamics of animal culture. The dark-eyed junco (*Junco hyemalis*) is a songbird with weak cultural song traditions because, in addition to learning songs socially, male juncos also invent or improvise novel songs. We compared songs shared by multiple males (i.e. socially learned) with songs recorded from only one male in the population (many of which should be novel) to gain insight into the advantages of social learning versus invention or improvisation. Song types shared by multiple males were on average of lower performance, on aspects of vocal performance that have been implicated in agonistic communication in several species. This was not explained by cultural selection among socially learned songs (e.g. selective learning) because, for shared song types, song performance did not predict how many males shared them. We discuss why social learning does not maximize song performance in juncos, and suggest that some songbirds may add novel songs to culturally inherited repertoires as a means to acquire higher-quality signals.

## 1. Background

Social learning facilitates coping with complex social or ecological challenges, and underlies animal culture in various taxa [1]. Social learning has costs too [2], such as time and energy dedicated to searching for suitable social models. Understanding these and other limitations should help explain when animals use social learning versus other forms of behavioural development (e.g. innate behaviour, learning with self [3]), and ultimately the emergence of animal culture.

One of the most specialized social learning systems in nature is that of oscine passerines (songbirds), who may use social learning to build song repertoires, target communication to individuals that share a song, or adjust signals to environmental conditions [4,5]. While oscines are ancestrally capable of social learning [6,7], some species develop normal song without social tutors (e.g. [8]), and others are flexible, learning some songs socially and acquiring novel songs by improvisation or invention (i.e. modification of tutor songs, or new songs altogether, respectively [4,9]). The latter is the case in the dark-eyed junco (*Junco hyemalis*).

**Figure 1.**
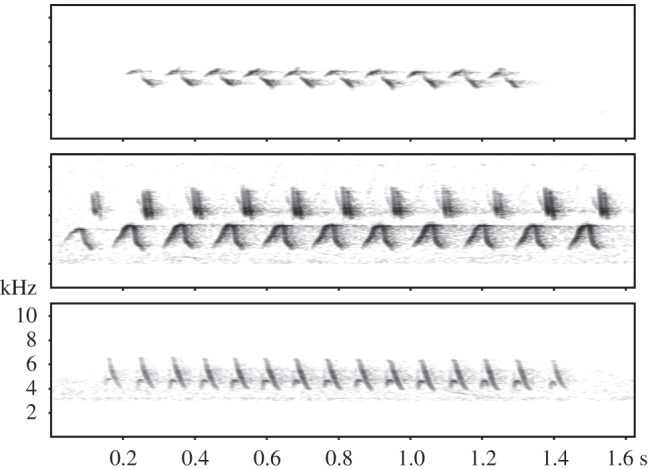
Spectrograms with examples of dark-eyed junco long-range songs, to illustrate the diversity of acoustic traits among song types.

Male dark-eyed juncos (hereafter, juncos) develop long-range song [10] (hereafter, song) during an early phase of socially stimulated singing that progressively crystallizes into distinct song types [11]. Many song types match the social stimuli young birds were exposed to during development, but many other song types appear novel, having been invented or improvised during development [11]. Song types consist of a syllable repeated to form a trill (figure 1), and adult males have a small repertoire of two to eight different song types [12,13]. A high incidence of novel song types is diagnosed by males not sharing songs with most neighbours [13], and by many song types being unique to individual males [12–14].

To gain insight into the advantages and limitations of social learning, we asked whether juncos' shared songs (i.e. recorded from multiple males, and therefore socially learned) differ from unshared songs. Unshared songs probably comprise a mixture of novel songs (improvised or invented during development) plus socially learned songs that, on average, are rarer and thus less likely to be recorded from multiple males. Thus, our comparisons can indicate either differences between socially learned versus novel songs, or that certain song traits are more effective at being socially transmitted or used (which are forms of cultural selection [15]). To assess the possibility of cultural selection among socially learned songs, we tested whether, among shared songs, song traits predict how many males sing them.

We focus our comparisons on metrics of song performance based on trade-offs among acoustic traits related to ventilation, repetition rate, sound amplitude or frequency modulation, for example, that suggest motor or physiological limitations (reviewed in [16,17]; in juncos [18,19]). In the junco, such metrics of performance do not appear to reveal male quality [20], but are instead a property of the different song types, and higher-performance song types are preferentially used during more motivated singing [19]. Similar aspects of song performance are related to aggressive singing in many other songbirds [21–30]. If shared songs are of higher performance, then social learning may be a means to obtain signals that are functionally of better quality. If, on the contrary, shared songs are of lower performance, this suggests limitations of social learning (e.g. low number of social models from which to choose songs), and helps explain why juncos invent or improvise novel songs during development.

## 2. Material and methods

We used an existing dataset of song measurements in over 1000 recordings of two dark-eyed junco populations (Oregon subspecies, *J. h. oreganus*) from southern California, USA (101 males in the University of California at San Diego, UCSD, and 50 males in Mount Laguna, ML), made during the breeding seasons of 2006 and 2007 [18,31]. These recordings comprise over 250 different song types sung in these populations, but were not designed to sample complete song repertoires of individual birds [19,20]. From the recorded songs, we computed four metrics of song performance: (1) *proportion of sound* and (2) *residual intervals*, which are metrics related to ventilation; (3) *vocal deviation*, which is related to motor performance; and (4) *predicted amplitude*, which reflects trade-offs between syllable complexity and relative sound amplitude [19]. We refer to Cardoso *et al*. [18,19] for detailed descriptions of acoustic measurements and metrics of performance, and for illustrations of song types differing in each performance metric, and below provide a summary of these metrics' rationale.

(1) *Proportion of sound* is a metric of performance based on the relation between the length of syllables and intervals. In some species longer syllables require longer intervals for mini-breaths (e.g. [32]), such that a higher proportion of sound in song (here calculated as the ratio of mean syllable length to the mean length of syllable plus inter-syllable interval in the trill) should indicate higher-performance songs in terms of ventilation. This is a simple metric of the type that has been used for various other songbird species (e.g. [28,33–35]).

(2) *Residual intervals* similarly quantify the length of intervals in relation to the airflow demands of syllables. In juncos, covariation between the length of syllables and length of intervening intervals is only apparent when controlling for syllable traits that may affect airflow [18]. Therefore, we use the residuals from the multiple regression of interval length on syllable traits in Cardoso *et al*. [18], as an improved way to assess airflow performance, with smaller residual intervals indicating higher performance.

(3) *Vocal deviation* was calculated as the distance to an upper limit of the relation between frequency bandwidth and repetition rate of syllables. In juncos, there is a strong negative upper boundary for the relation on how rapidly birds can repeat a syllable and how widely they can modulate its sound frequency [18,36]. Vocal deviation was calculated as the orthogonal distance from this upper boundary, such that smaller vocal deviations indicate higher performance (i.e. songs approach the upper boundary).

(4) *Predicted amplitude* uses the trade-off between sound amplitude and syllable complexity, as evaluated from changes in relative amplitude when juncos switch song types. It was calculated as the predicted value from the multiple regression of relative sound amplitude on syllable traits in Cardoso *et al*. [18]. The objective of computing predicted amplitude is not to estimate at which amplitude a song type is to be sung because, as in other species, song amplitude is very labile in juncos [37,38], but to infer differences in vocal demands when juncos are singing different song types at the same amplitude. Smaller values indicate higher performance, in that song types with lower predicted amplitude (i.e. with traits inferred to lower amplitude) should be more demanding to sing at a given, high amplitude.

These metrics of performance have been shown before to be highly repeatable within the same junco song type, even if sung by different males, but weakly or not repeatable within the repertoires of individual junco males [19]. Thus, song performance in juncos is a property of the song types, and we use each song type present in the population as the statistical unit for analyses. We averaged measurements across recordings of the same song type and male, and then across all males singing that song type in each population (UCSD or ML). The electronic supplementary material contains the full dataset of song measurements and performance metrics used in this study.

We compared song performance between song types recorded by multiple males versus those recorded from a single male in the population using a general linear model (GLM). Population of origin (UCSD versus ML) is an additional factor in the GLM, to control for possible population differences in song performance. Because some song types recorded from a single male may be shared but not recorded from the other males, this is a conservative test for differences between shared and non-shared song types. Results using metrics of song performance are best interpreted by consideration of similar analyses on the raw acoustic traits (e.g. syllable length, frequency bandwidth, etc.) used in their computation. Therefore, to aid the interpretation, results in table 1 replicate the above analysis for every raw acoustic trait measured.

**Table 1.** Results of general linear models comparing traits of song types recorded from a single versus multiple males, in UCSD versus ML. Statistically significant results are marked in bold. Underlined indicates higher means for song types recorded from a single male or in UCSD.

	recorded from 1 versus more than one male	population effect (UCSD versus ML)
acoustic traits of syllables	*F*_1,280_ (*p*)	*F*_1,280_ (*p*)
length of syllables	**3.903** **(0.049)**	1.311 (0.253)
length of inter-syllable intervals	**4.736 (0.030)**	0.018 (0.893)
syllable rate	1.469 (0.226)	1.086 (0.298)
peak frequency	0.886 (0.347)	1.813 (0.179)
frequency bandwidth	**7.922** **(0.005)**	**10.040 (0.002)**
minimum frequency	2.940 (0.088)	**54.841** **(0.000)**
maximum frequency	**4.904** **(0.028)**	1.129 (0.289)
number of frequency inflections	0.027 (0.870)	0.739 (0.391)
number of elements	1.658 (0.199)	0.071 (0.791)
length of two voices	1.377 (0.242)	**5.793** **(0.017)**
length of ‘rattles’	0.077 (0.782)	0.177 (0.675)
length of harmonics	3.094 (0.080)	0.113 (0.737)
length of intra-syllable gaps	1.415 (0.235)	0.978 (0.323)

To account for the possibility that differences in performance between song types sung by multiple or single males are due to cultural selection (preferential learning or the use of song types depending on their performance), within each population we regressed the mean performance of each shared song type (i.e. songs that we are confident were learned socially) on the number of males singing them. We then used the predicted performance for song types sung by only one male, from these regressions, as null hypotheses for comparison with the real performance of song types recorded from only one male, using one-sample *t*-tests. All tests were run in SPSS v. 21.

## 3. Results

**Figure 2.**
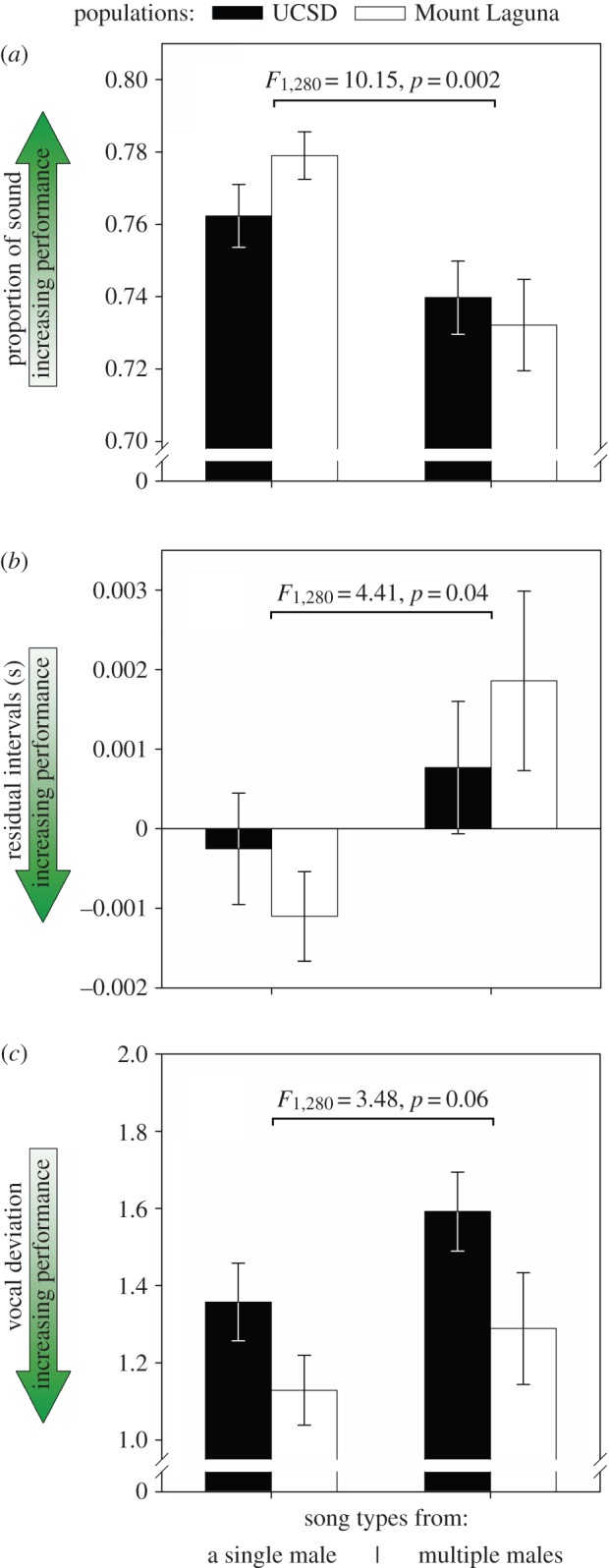
(*a*–*c*) Differences in three metrics of performance between song types recorded from a single or multiple males. Populations are represented by different colours, means and standard errors are shown, and the direction of increasing performance is indicated by arrows.

Forty-five per cent of song types recorded in UCSD (75 out of 168) and 25% in ML (29 out of 115) were shared among two or more males; the remaining were recorded from one male each (see the electronic supplementary material). Song types recorded from only one male were on average of higher performance in *proportion of sound* (main effect: *F*_1,280_ = 10.15, *p* = 0.002; population effect: *F*_1,280_ = 0.88, *p* = 0.35; figure 2*a*) and *residual intervals* (main effect: *F*_1,280_ = 4.41, *p* = 0.04; population effect: *F*_1,280_ = 0.08, *p* = 0.77; figure 2*b*). These results were driven by synergistic differences in the most important traits used by these metrics (length of syllables and of intervals between syllables; table 1).

Song types recorded from only one male also tended to have better *vocal deviation* (main effect: *F*_1,280_ = 3.48, *p* = 0.06; figure 2*c*), and ML song types also had on average better vocal deviation than UCSD (population effect: *F*_1,280_ = 5.28, *p* = 0.02). Of the two acoustic traits used to compute *vocal deviation* (syllable rate and frequency bandwidth), only frequency bandwidth contributed to these results (table 1). The population difference in *vocal deviation* was also solely due to frequency bandwidth: minimum frequency was higher and, consequently, frequency bandwidth was lower in the urban UCSD population than in ML (table 1), a result previously reported for these populations and probably related to anthropogenic noise [31,39].

*Predicted amplitude* did not differ between song types recorded from a single or multiple males nor between populations (main effect: *F*_1,280_ = 0.16, *p* = 0.69; population effect: *F*_1,280_ = 0.03, *p* = 0.86).

Among song types recorded from multiple males, no metric of performance predicted the number of males singing them (absolute values of all standardized regression coefficients less than or equal to 0.18, all *p* ≥ 0.14; table 2). We also visually examined plots of number of males singing each song type versus song type performance, and did not detect suggestive trends for nonlinear relations. Despite no significant evidence for cultural selection based on song performance, we nonetheless compared the performance of song types recorded from single males with the predicted values from the above regressions, to test whether their higher performance could be expected from the estimated (though non-significant) trends of cultural selection. Conclusions were similar to the earlier analyses: for *proportion of sound*, *residual intervals* and *vocal deviation*, the performance of songs recorded from single males differed from the value predicted by cultural selection in at least one population (table 2), and the metric *predicted amplitude* did not differ from the expected value for songs recorded from single males (table 2).

**Table 2.** Linear regressions of performance on number of males singing shared song types, and one-sample *t*-tests of the performance of song types recorded from only one male versus the predicted value of performance from these linear regressions. Statistically significant results are marked in italic. Sample sizes are 75 shared and 93 non-shared UCSD song types, and 29 shared and 86 non-shared ML song types.

	linear regressions		one-sample *t*-tests	
metric of performance	UCSD	ML	UCSD	ML
proportion of sound	*β*_st_ = −0.05 (*p* = 0.65)	0.10 (0.61)	*t*_92_ = 1.98 (*p* = 0.051)	*t_85_ = 9.46* (*p* < *0.001*)
residual intervals	0.08 (0.50)	0.004 (0.98)	−0.50 (0.62)	−*5.17* ( < *0.001*)
vocal deviation	−0.17 (0.14)	−0.14 (0.47)	−*4.12* (<*0.001*)	−*4.50* (<*0.001*)
predicted amplitude	0.07 (0.58)	−0.006 (0.97)	1.72 (0.09)	−1.31 (0.20)

## 4. Discussion

Shared songs were on average of lower performance than songs recorded from only one male, many of which should have been invented or improvised during development. We found no evidence that these differences were driven by cultural selection based on song performance, because the estimated cultural selection trends were small and not significant, and because differences between shared and non-shared songs persisted after accounting for those estimated cultural selection trends. Instead, our results suggest that male juncos invent or improvise some song types during development that are, on average, higher performance than the songs they learn socially.

Junco song types with higher performance—specifically with shorter intervals and syllables, and faster syllable rate—are preferentially used during more motivated singing (i.e. when observed singing longer songs, for example because of counter-singing with neighbours [19]), and similar aspects of song performance are related to aggressiveness in many other species [21–30]. It is possible, but not yet tested in juncos, that song performance is also relevant for mate attraction. Because we found that non-shared songs in juncos are, on average, of higher performance, invention or improvisation of song may be a means to add functionally relevant signals to their repertoire. This is clearer for the performance metrics related to ventilation (*proportion of sound* and *residual intervals*), as our results with those metrics were due to synergistic differences in multiple traits (lengths of syllables and of inter-syllable intervals). Our results with the metric *vocal deviation* are less clear to interpret functionally, because they were driven by a single trait, frequency bandwidth, while it was previously shown that the relation between *vocal deviation* and motivation is instead due to syllable rate [19].

Our set of performance metrics is not exhaustive. There may be additional trade-offs among song traits not captured by these metrics, and different aspects of song performance exist that could also differ among song types, such as consistency across song renditions [40], the occurrence of mistakes during singing [41], or several aspects of singing output and effort (e.g. song duration, rate or absolute amplitude). Other aspects of song performance may show different, or even opposed patterns to the ones showed here. Notwithstanding that there are many unstudied aspects of performance, the functional link between some of the performance metrics we studied and signalling motivation [19,21–30] indicates that the higher performance of novel songs is relevant for communication.

There are several possible explanations for our finding that juncos do not preferentially learn and use the higher-performance songs in their population. (i) Song learning may be guided by other functional criteria, such as proximity [42], song type commonness [43], or specific types of social interactions [44]. (ii) It has been suggested that differences in song performance may be better perceived when different individuals sing the same song type, and that some individuals may benefit from sharing songs to advertise their singing ability, while others benefit from not sharing to avoid being negatively evaluated [45]. Song type sharing is very low in juncos [13], but if receivers nonetheless compare the performance of shared song types, then perhaps many individuals benefit from not sharing the highest-performance song types in the population, where they could be negatively evaluated. (iii) Another explanation might be that the acoustic diversity among social models of long-range songs is lower than among non-social models (e.g. own sounds generated during song development). Namely, juncos have a distinct category of short-range song used during courtship [46] that contains a great diversity of syllables sung continuously [10]. Among this diversity of sounds in short-range song, some may be appropriate models from which to develop long-range songs, thus facilitating the emergence of novel, high-performance songs.

The input of high-performance song types into the cultural pool of junco populations must be counterbalanced by some mechanism, because mean song performance cannot increase indefinitely. Three non-mutually exclusive cultural phenomena could compensate for the input of high-performance songs. (i) Negative cultural selection on song performance, whereby high-performance song types are less likely to be learned and sung by other males (e.g. the scenario discussed above, where individuals avoid sharing high-performance song types). Although we did not find evidence for negative cultural selection of higher-performance song types, we cannot exclude that weak cultural selection takes place that, in the long run, maintains mean song performance at equilibrium. (ii) Random cultural drift, whereby rare song types are more likely to be lost from the population irrespective of their performance. Because each invented or improvised song type is very rare by definition, they should be more affected by random loss, thus reducing the effect of novel, high-performance songs on mean song performance of junco populations. (iii) Progressive decrease of performance due to cultural mutation, such as changes during song learning. Junco songs can accumulate changes over the generations [31], and it was shown in a related species that changing song in the direction of increased performance disrupts copying accuracy of syllables [47]. Therefore, perhaps changes during learning are not random in relation to song performance, and tend to reduce rather than increase performance to preserve copying accuracy.

The input of novel songs every generation also makes it difficult to sustain long-term cultural traditions, such that junco populations have high levels of song diversity [48]. In turn, while in other species receivers may prefer locally common songs (reviewed in [49]), the song diversity within junco populations should protect novel songs from being discriminated against by receivers (inasmuch as novel songs conform to typical acoustic traits in the population [50,51]), facilitating the coexistence of socially learned and novel songs.

In conclusion, our finding that shared junco songs are on average of lower performance points to a greater degree of versatility during song development than currently realized. It is known that songbirds can acquire or retain socially learned songs selectively (reviewed in [52]) or improve defective socially learned songs [53,54]; our results further suggest that some species can also add novel, high-performance songs to their repertoire. This augments the set of options that developing songbirds may use to acquire good-quality signals.

## Supplementary Material

Dataset of song measurements and performance metrics used in this study.

## Data accessibility

The dataset supporting this article was uploaded as the electronic supplementary material.
